# Natural Killer Cell Diversity in Viral Infection: Why and How Much?

**DOI:** 10.20411/pai.v1i1.142

**Published:** 2016-08-31

**Authors:** Catherine A. Blish

**Affiliations:** 1 Department of Medicine, Division of Infectious Diseases and Geographic Medicine; 2 Immunology Program, Stanford University School of Medicine, Stanford, California

**Keywords:** NK cell or natural killer cell, virus, diversity, repertoire, lymphocyte, mass cytometry

## Abstract

Natural killer cells are a diverse group of innate lymphocytes that are specialized to rapidly respond to cancerous or virus-infected cells. NK cell function is controlled by the integration of signals from activating and inhibitory receptors expressed at the cell surface. Variegated expression patterns of these activating and inhibitory receptors at the single cell level leads to a highly diverse NK cell repertoire. Here I review the factors that influence NK cell repertoire diversity and its functional consequences for our ability to fight viruses.

## INTRODUCTION

Natural killer (NK) cells are innate lymphocytes that can rapidly eliminate infected or tumor cells and modulate immune responses through the secretion of cytokines and chemokines. First identified in mice and humans in the 1970s on the basis of their ability to kill tumor cells, they form a critical first line of defense, capable of acting within minutes and without the need for priming [1, 2]. NK cells are now recognized to be part of the larger family of innate lymphoid cells (ILCs) [3], though this review will focus exclusively on “classical” NK cells in humans. A single NK cell can act as a serial killer—engaging multiple targets in sequence over a rapid time scale [4]. NK cells may help prevent cancer; individuals with high NK cell activity are less likely to develop cancer [5]. The important role of NK cells in cancer recognition, coupled with their remarkable speed and potency, has led to excitement about the development of immunotherapies harnessing NK cells to attack cancer [6–9].

The importance of NK cells for fighting viral infections was revealed by Biron and colleagues' description of severe herpes virus infectious in NK cell-deficient individuals [10]. The association between NK cells and viral susceptibility has held true for additional immunodeficiencies in which NK cell dysfunction is a prominent feature, including X-linked lymphoproliferative syndrome (XLP) and X-linked immunodeficiency with Mg^2+^ defect, EBV infection, and neoplasia (XMEN) [11–16]. These findings, combined with the recent descriptions of NK cells with memory-like capacity [17–22], has raised the possibility that NK cells might be an important component of new antiviral vaccine or immunotherapy strategies. However, for these vaccine or immuno-therapy approaches to be a success, we must first understand which NK cells to harness and how to best tune their activity to attack the appropriate pathogen. This requires consideration of the diversity of the human NK cell repertoire, and how, within that diverse repertoire, we can select the NK cell activities we desire.

## NK CELLS: A DIFFERENT KIND OF DIVERSITY

Diversity is an intrinsic and critical characteristic of the immune system, aiding in our ability to recognize and eliminate a wide variety of potential pathogens. Diversity is often attributed primarily to T and B cells, lymphocytes that somatically rearrange their antigen-specific receptors to provide billions of potential specificities [23]. During B and T lymphocyte development, there are checks and balances in place to avoid autoreactivity while generating flexibility and a vast array of specificities. Natural killer cells, on the other hand, do not have somatically rearranged antigen-specific receptors. They are therefore tasked with responding to the vast array of potential pathogens through germline-encoded receptors, requiring different pathways to provide a response that is rapid, tunable, and self-tolerant.

To achieve this, NK cells generate diversity through two primary mechanisms: the first based on genetic diversity within killer immunoglobulin-like receptor (KIR) genes, and the second based on the assortment of receptors at the cell surface. In humans, KIR are the second most polymorphic genes after the human leukocyte antigen (HLA) genes, and individuals differ in their KIR gene content [24, 25]. As a result, KIR genes are a major driver of NK cell diversity, which will be summarized only briefly here. KIRs consist of two major types of receptors, the inhibitory KIR (denoted by an “L” in the name for a long cytoplasmic tail), and the activating KIR (denoted by an “S” for a short cytoplasmic tail). Individuals can be sorted into two groups based on their KIR genotypes. The KIR A group has primarily inhibitory KIR and either no or one activating KIR, while group B has additional activating KIR genes [25]. Inhibitory KIRs recognize HLA, sending an “all-clear” signal to prevent the NK cell from lysing the target cell. As a result, KIR are a critical component of NK cell function, and NK cells are educated (also called licensed or armed) through their KIR to be exquisitely sensitive to perturbations in HLA expression [26–28]. As KIR and HLA are inherited independently, KIR-expressing NK cells are educated if the KIR encounters its cognate HLA ligand during development, or uneducated if the HLA ligand for the KIR is absent. The importance of KIR diversity for the NK cell repertoire and its responsiveness has been studied and reviewed extensively [22, 25, 29–31]. In particular, a significant body of work points to the importance of balancing selection in maintaining divergent KIR haplotypes that favor either survival from infectious disease (during times of epidemics) or reproduction (to favor recovery) [24, 32]. The critical balance between reproductive needs and protection from infection is beautifully highlighted in a series of studies demonstrating how different maternal and paternal KIR/HLA combinations influence reproductive outcome [22, 24, 33–38]. These findings, and many others, establish the critical importance of genetics in maintaining NK cell diversity.

Rather than reiterate the importance of the genetic contributions to NK cell diversity, in this review I seek to focus instead on the second major contributor to NK cell diversity: how the assortment of receptors at the cell surface generates NK cell diversity. The phenotypic diversity of NK cells is based upon combinatorial expression patterns of activating and inhibitory natural killer receptors (NKRs), including KIR and many other genes, with each NK cell capable of expressing a different combination of NKRs. Since NK cells integrate signals from this array of activating and inhibitory signals, this phenotypic diversity has significant implications for NK cell function. For instance, a NK cell with a large number of activating NKRs may be more readily triggered. Thus, the phenotype and function of NK cells are tightly linked.

## THE MAGNITUDE OF HUMAN NK CELL DIVERSITY

The idea of subsetting NK cells based on phenotypic markers was first proposed by Lanier and colleagues in 1983 [39], and since then the number of populations and recognition that they can perform distinct functions has steadily increased [40–43]. NK cells are traditionally divided based on CD56 and CD16 expression, with CD56^bright^CD16^-^ NK cells thought to be relatively immature and specialized for cytokine secretion while the more abundant CD56^dim^CD16^+^ NK cells are fully mature and primed for killing. In fact, there is significant functional overlap between these groups, and intermediate populations of CD56^-^CD16^-^ NK cells can also arise, particularly in the setting of chronic infection [44].

In light of their critical importance in distinguishing self from “altered self,” several studies have elegantly explored the expression patterns of the major inhibitory receptors: inhibitory KIRs, NKG2A, and LILRB1 [45–50]. These studies demonstrated that NK cells express every possible combination of the inhibitory receptors they encode, resulting in NK cell subsets with one or several inhibitory receptors as well as a significant subset of NK cells lacking inhibitory receptors. Further, the education status of these NK cells varies based on the combined KIR and HLA geno-types, conferring a distinct functional capacity to these NK cell subsets [45–50].

More recently, the advent of cytometry by time-of-flight (CyTOF, also called mass cytometry) [51, 52], has allowed us to generate a more complete understanding of the diversity of receptor expression patterns on NK cells that includes the activating receptor profiles [50, 53–55]. CyTOF is a flow cytometry platform that uses metals instead of fluorophores to tag antibodies, with the readout by mass spectrometry, and thus allows the use of ~40 parameters simultaneously without the need for compensation [51, 52]. CyTOF profiling of the healthy human NK cell repertoire revealed between 6,000 and 30,000 unique NK cell subsets, based on combinatorial expression patterns of NKR, per individual [50]. As these subsets were not necessarily shared between individuals, more than 120,000 NK cell subsets were present in the 22 individuals studied [50]. The magnitude of this diversity was surprising; studies of the receptor profiles in twins revealed both genetic and environmental influences. Inhibitory receptor profiles were genetically determined, but the activating receptor expression patterns appeared to be under environmental influence [50]. The result is a highly diverse and adaptable repertoire that is quite distinct even among adult twins, but maintains self-tolerance through strict regulation of inhibitory receptor expression patterns [50].

An important consideration in quantifying NK cell diversity is that these calculations are highly dependent on the number of markers and methods used. For instance, the diversity score is diminished if fewer markers are used or if the markers selected are either highly and universally expressed or expressed at a very low frequency. Thus, while these methods are very valuable to compare populations within a given study, comparison between studies requires that identical markers be used. A second consideration is the fact that any single diversity score does not fully capture the repertoire characteristics and distributions. For example, the Inverse Simpson Index is commonly used to quantify both immune repertoire and microbiome diversity because it is adaptable to count data and does not require normally distributed data. However, this index can result in the same value with very different population structures—which may have significant functional implications. A specific example of this is that two healthy adults have nearly identical NK cell diversity scores by the Inverse Simpson Index (123 and 126), but a very different number of NK cell subsets among the same number of total NK cells (2189 and 446), indicating that the distribution of the populations must be quite different (Simpson and Blish, unpublished). Thus, while diversity calculations provide a convenient method to quantify and understand the NK cell population structure, their limitations must be understood as well. With these limitations in mind, it is still important to understand the factors that control the development, maintenance, and function of this diverse NK cell repertoire.

## THE IMPACT OF IMMUNE EXPERIENCE ON THE NK CELL REPERTOIRE

Some clues about the factors that control the development and maintenance of NK cell diversity have come from studies of age-related changes in the NK cell repertoire (reviewed in [56]). Aged individuals have decreased expression of NKp30, NKp46 and NKG2D, and NKG2A and increased expression of KIR, LILRB1, and TIGIT [57–61]. Some studies suggest that NK cells maintain their cytolytic function with aging [57, 58], while others suggest that cytotoxicity and cytokine production decrease with aging [62]. The extent to which age-related changes are due to intrinsic aging vs. accumulated environmental exposures, including cytomegalovirus (CMV) infection, are not entirely clear. However, while CMV infection is clearly a driver of many of these changes [63], some of these changes are not driven solely by CMV. Regardless of age and CMV status, “experienced” CD57^+^ NK cells express more NKRs per cell and significantly increase expression of a distinct pattern of activating and inhibitory NKR [53]. CD57^+^ NK cells are also skewed towards cytokine production at the cost of cytotoxicity [54]. These data suggest that “immune experience” may be a better metric for NK repertoire perturbations than aging itself. Consistent with this idea, in healthy adults, NK cell diversity significantly correlates with expression of CD57, but not with age, suggesting that immune experience is a major driver of NK cell diversity [54]. The idea that immune experience shapes NK cell repertoire diversity is also supported by the fact that cord blood NK cells have low CD57 expression and significantly lower NK cell diversity than adult NK cells [54]. Finally, short-term *in vitro* exposure to viruses (HIV-1, West Nile Virus [WNV]) augments NK cell diversity [54], suggesting that serial viral exposures might shape and diversify the NK cell repertoire. Thus, if a wide range of receptor profiles accumulates immune experience, it is interesting to consider whether different NKR, singly or in combination, are particularly important in the response to different viruses.

## ROLE OF SPECIFIC RECEPTORS AND COMBINATIONS IN DIFFERENT VIRAL INFECTIONS

One explanation for the generation of NK cell diversity during an acute antiviral response is that the repertoire is adapted to generate a range of specificities in order to find the right “solution” for each virus. Along these lines, it stands to reason that a variety of different NK cell receptors might contribute to the recognition of any given virus, quite possibly with complementary and overlapping functions. Consistent with this idea, many different studies have identified the role of particular NK cell receptors in the response to different viruses. I have summarized these findings in Table 1. They represent a mixture of epidemiologic associations and mechanistic studies. As even this exhaustive list does not comprehensively assess all of the literature, I also refer the reader to excellent reviews on the role of natural cytotoxicity receptors (NCRs) and NKG2D in responding to multiple viruses [64–68] and recent reviews of the role of KIR and their evolution in disease [69, 70].

**Table 1.** NK cell receptor virus interactions

Virus	NK Cell Receptor	Brief Description	Reference
CMV	NKG2C	NGK2C^+^ cells expand during CMV infection	[106–112, 119, 120, 132, 133]
CMV	LIL-11, LIR-1	UL18 inhibits LIR-11;activates LIR-1	[134]
CMV	NKp30	NKp30 inhibited by pp65	[135]
CMV	NKG2D	Several viral proteins bind NKG2D to limit recognition	[68]
Influenza	2B4 and NTB-A	2B4 and NTB-A receptors bind the influenza viral hemagglutinin and co-stimulate NK cell cytotoxicity.	[98]
Influenza	KIR2DL3	KIR2DL3 and KIR3DL1 and HLA-C1 homozygosity leads to have enhanced IFN-γ secretion and degranulation to influenza A infection in vitro. Individuals with KIR3DL1/S1 or KIR2DL1 but lacking the ligand enriched among ICU patients during the 2009 flu pandemic, as were individuals with KIR2DL2/L3 and its cognate ligand.	[92, 93]
	KIR2DL1		
	KIR3DL1/S1		
Influenza	NKp46	NKp46 interaction with HA leads to infected-cell lysis, with potential for escape of this pathway by NA-mediated removal of sialic acid residues from NKp46 to decrease recognition	[94–96] [97]
Influenza	NKG2D	NKG2D (and NKp46) mediated recognition of influenza-infect-ed dendritic cells	[95]
HIV	CD94/HLA-E	CD94/HLA-E interaction may contribute to NK cell dysfunction in HIV infection	[136]
HIV	FcRγ	FcRγ^-^NKp30^-^NKp46^-^ NK cells are expanded in HIV and have enhanced ADCC activity	[137]
HIV	KIR2DS4	Full-length KIR2DS4 associated with disease progression	[138]
HIV	KIR3DS1/KIR3DL1	Combinations of KIR3DS1 and/or KIR3DL1 and HLA-Bw4-80I are associated with delayed HIV progression. KIR3DL1 and HLA-B density and binding alter education and HIV responsiveness; KIR3DS1^+^ NK cells expand and can kill HIV-infected cells	[72, 73, 75–77, 79, 87, 139–141]
HIV	KIR2DL1-3^+^	KIR2DL1-imprinting on HIV strains; KIR2DL1-3^+^ NK cells more responsive	[142, 143]
HIV	KIR2DL3	NKG2A^+^KIR2DL3^+^ cells potently secrete CC-chemokines, particularly in HLA-C2 individuals and KIR2DL3 is associated with resistance to HIV acquisition in HIV-exposed babies; selection of p24 sequence associated with KIR2DL3 escape	[91, 144–146]
HIV	LILRB1	LILRB1^+^ NK cells control HIV-1 replication in DCs	[147]
HIV	NCRs	NCRs are decreased in chronic HIV infection	[148]
HIV	KIR	Nef induces endocytosis of HLA-I molecules, helping virus escape from NK cells	[149]
HIV	NKG2D	Nef downregulated NKG2D ligand in infected cells causing decreased cytotoxicity	[150]
HIV	NTB-A, UL-16BP	vpu/nef downregulate NK cell ligands: NTB-A, UL16-BP	[151]
HIV	NKG2A	NKG2A^+^ NK cells respond more frequently than NKG2A- to HIV^+^ T cells; based on a conserved HIV-1-dervired peptide presented by HLA-E that renders cells susceptible to NKG2A	[89, 90]
HIV	NKG2D	NKG2D acts as a co-receptor for natural killer cell-mediated anti-HIV-1 antibody-dependent cellular cytotoxicity.	[152]
HIV	NKG2D/NKp46	Lysis of HIV-1-infected autologous CD4^+^ primary T cells by interferon-alpha-activated NK cells requires NKp46 and NKG2D.	[153]
HIV	NKp46 NKp30	NKp30 and NKp46 expression correlates with AIDS-status of successfully treated patients	[154]
HIV	NTB-A	Vpu downregulates NTB-A in infected T-cells, causing decreased degranulation by NK cells	[155]
HIV	Siglec-7	Siglec-7 is decreased in NK cells of viremic patients	[156]
HIV and other pathogens	DNAM-1 and NKG2D	Review on NK-T crosstalk mediated by DNAM-1 and NKG2D and their ligands, in the context of infections	[67]
HIV/HCV and other pathogens	NCRs	Reviews on NCRs and pathogen interactions	[65, 66]
HCV	KIR2DL2/L3	KIR2DL3/L3 increases function; KIR2DL3/HLA1C1 is associated with response.	[99–101]
HSV-2	NKG2C, KIR, CD57	HSV-2 infection drives NKG2A^-^NKG2C^+^KIR^+^CD57^+^ NK cells	[157]
HSV, VSV	NKG2D	HSV decreases MICA, ULBP1, ULBP2, ULBP3	[158]
KSHV	LFA, others	K3 and K5 viral proteins downregulate MHC class I molecules, ICAM-1 ad B7-2, ligands for NK cell-mediated cytotoxicity receptors	[159]
Hantavirus	NKG2C	NK cells expressing NKG2C expand (though most subjects also CMV^+^)	[160]
Multiple viruses	NKG2D and NCR	Review summarizing data from multiple viruses mwith methods to decrease NKG2D and possible NCR ligands	[64]
CHIKV	NKG2C and CD57	Mature cells more responsive	[161]
Dengue	Inhibitory KIRS	NK cells with inhibitor KIRs respond preferentially to DENV	[161, 162]
WNV and Dengue	NKp44	NKp44 directly binds to purified DV and WNV envelope proteins. Interaction of NK cells with infective and inactivated WNV results in NKp44-mediated NK degranulation	[163]

## NK CELL RECEPTORS AND HIV

Perhaps one of the most studied interactions between specific NK cell receptors and a virus is the interaction between KIR3DS1/L1 and HIV. In fact, the influence of KIR3DS1 on disease, particularly HIV, has recently been the topic of an entire review [71]. This association came to light based on the discovery that HIV-infected individuals with both KIR3DS1 and the HLA-B alleles containing the Bw4-80Ile epitope experience slower progression to AIDS [72, 73]. An additional study found that the combination of KIR3DL1 and HLA-Bw4-80I was also associated with slower disease progression [74]. Further confirming the importance of NK cell expression of KIR3DS1/ L1/HLA-Bw4-80I, copy number variation in KIR3DS1 and KIR3DL1 are associated with the HIV set point viral load, but only in the presence of the Bw4-80I allele [75]. KIR3DS1/L1 alleles are also associated with lower risk of HIV transmission between partners [76].

Consistent with these epidemiologic associations, NK cells expressing both KIR3DS1 and KIR3DL1 expand during HIV infection, but only in the presence of the HLA Bw4-80I allele [77]. The ability to suppress viral replication *in vitro* was associated with KIR3DS1 or KIR3DL1 and HLA-Bw04 expression [77, 78]. In addition, individuals with protective KIR3DL1/S1 geno-types inhibited HIV replication more potently than those lacking such alleles through secretion of CC-chemokines [79]. The antiviral efficacy of KIR3DS1 may relate to its association with the ITAM-bearing receptor DAP12 [80]. In all of these studies, it is important to note that many of the effects of KIR3DS1 vs. KIR3DL1 are difficult to dissect, as most KIR3DS1^+^ individuals also express KIR2DL1. In addition, KIR3DL1 is the most diverse of all the KIR, and different allotypes have dramatically different effects on HLA binding [81]. Taken together, both epidemiologic and experimental data suggest that both KIR3DS1 and KIR3DL1 play a role in the response to HIV, yet it is not apparent how both an activating and inhibitory receptor, that are nearly identical in their extracellular domain, might both enhance responses to the same pathogen.

Recent data may provide some insight into this potential conundrum. The first issue is whether KIR3DS1 and KIR3DL1, given their similar extracellular domains, truly bind to the same ligand (in which case it would be hard to reconcile their similar effects in light of their opposing roles on NK cell activation). The inhibitory receptor KIR3DL1 binds to HLA-B molecules containing the Bw4-80Ile epitope [82]; however, several studies have failed to demonstrate similar binding for the activating KIR3DS1 receptor to Bw4-80I [80, 83, 84]. One potential limitation of these negative data, however, is that the researchers did not study HIV-infected cells, and it is possible that HIV peptides might alter the ability of KIR to bind HLA. Consistent with this idea, O'Connor et al. recently demonstrated that two different HIV peptides allow binding of KIR3DS1 to Bw4 alleles [85]. Furthermore, a recent study demonstrated that KIR3DS1 binds to open conformers of HLA-F [86], indicating that even if it binds Bw4, it has additional ligands. Synthesizing these studies, it appears likely that there are multiple pathways by which KIR3DL1 and KIR3DS1 can contribute to HIV responses. The first, which explains the effects of KIR3DS1, is that NK cells bearing KIR3DS1 become activated through direct recognition of either HLA-F open confomers or of HLA-Bw4 alleles with specific HIV peptides. The second pathway, explaining the contributions of KIR3DL1, involves the effects of KIR3DL1/HLA-Bw4 on educating NK cells—leading to a generalized high activation status. As individuals with KIR3DL1 and the Bw40-80I epitope have highly educated NK cells, they are better able to suppress HIV replication, consistent with recent findings that KIR3DL1 and HLA-Bw4 density significantly influence HIV replication [87]. Thus, two entirely different pathways—one activating and associated with direct recognition, and the other inhibitory but associated with better “arming” NK cells through education/licensing— might contribute to HIV responses. This finding also stresses the importance of diversity within the NK cell response—in this case two different solutions, generated by distinct and overlapping subsets of NK cells—are available to respond to HIV-infected cells.

Of course, recognition of HIV-infected cells is not just associated with KIR3DL1/S1 and HLABw4. Multiple interactions between KIR and HIV have been documented [88], as shown in Table 1. NKG2A^+^ NK cells respond more frequently to HIV-infected cells than do NKG2A^-^ NK cells [89]. The recent study by Davis et al., provides a potential mechanistic explanation for why this inhibitory receptor might contribute to HIV recognition [90]. The authors demonstrate that a highly conserved HIV peptide presented by HLA-E renders the cells susceptible to NKG2A-mediated killing (presumably by abolishing the recognition and preventing inhibitory signaling) [90]. Thus, NKG2A-expressing NK cells, which relatively infrequently co-express KIR, are not inhibited by the HLA-E on the surface of HIV-infected cells, whereas the more highly educated KIR-expressing NK cells will be inhibited through recognition of HLA-C that remains highly expressed during HIV infection. Consistent with this idea, another recent study demonstrated that KIR2DL3^+^NKG2A^+^ NK cells potently responded to HIV-infected cells though secretion of CC chemokines. But this effect was primarily seen in KIR2DL3^+^ individuals lacking HLA-C2 [91]. The “educated” KIR3DL2-expressing cells in HLA-C1 homozygotes might be inhibited by the HLA-C that is retained on the surface of HIV-infected cells. Finally, a variety of activating receptors, including natural cytotoxicity receptors, NKG2D, KIR2DS4, FcRγ, NTB-A, and an additional inhibitory receptor, LILRB1, are all associated with HIV responses (Table 1). These data provide further support for the idea that NK cells have evolved diverse mechanisms to recognize and respond to HIV infected cells, making diversity in receptor expression an intrinsic characteristic of NK cells responding to viruses.

## DIVERSE NK CELL RECEPTORS ARE INVOLVED IN THE RESPONSE TO OTHER VIRUSES

Specific KIR have also been associated with influenza infection. Expression of KIR2DL3 and KIR3DL1 was associated with more robust IFN-γ and cytolytic responses *in vitro* [92]. A more complex picture was observed in a clinical study in which either KIR3DL1/S1- or KIR2DL1-expressing individuals lacking the ligand or KIR2DL2/L3 and its cognate ligand were enriched among ICU patients during the 2009 influenza pandemic [93]. In addition to these KIR associations, there are well documented examples of natural cytotoxicity receptors, NKG2D, 2B4, and NTB-A playing a role in the recognition of influenza-infected cells [94–98], which are summarized in Table 1.

Along similar lines, NK cells expressing KIR2DL3/L3 have increased degranulation to hepatitis C (HCV) [99]. In addition, the compound genotype KIR2DL3 homozygosity and HLA-C1 is associated with HCV responses [100]. This observation may be explained by the fact that HLA-E expression was significantly unregulated in HCV-infected patients, but that KIR2DL3^+^NKG2A^-^ NK cells were not susceptible to HLA-E mediated inhibition and therefore preserved their function [101]. Many additional associations with antiviral responses are noted and summarized in Table 1. In particular, NKG2D is a recurring mediator of recognition for multiple tumor and infected cells. The fact that viruses have evolved a variety of means to downregulate NKG2D ligands provides evidence for its importance in mediating NK cell responses to a variety of pathogens [68, 102]. It is also important to note that NKG2D is the focus of much research in large part because its ligands are known. However, for many other NK cell activating receptors, the ligands remain unknown. As a result, there might well be other escape mechanisms that we do not fully understand or appreciate.

## THE DRAMATIC INFLUENCE OF CMV ON THE REPERTOIRE

While I have touched briefly above on the specific NKRs involved in the recognition of CMV-infected cells, the impact of CMV infection on the NK cell repertoire is so dramatic that it has been the subject of several prior excellent reviews that do the subject far more justice than I can here [63, 103–105]. The most obvious impact of CMV infection is the expansion of a NKG2C^+^ NK cell subpopulation in a subset of CMV-seropositive individuals [106–112]. This NK cell subpopulation is additionally characterized by high expression of CD57, low expression of NKp30, CD161, NKG2A, and Siglec-9 and often expression of self-specific KIR, suggesting that the expansion of these cells is restricted to an educated subset of NK cells [107, 113, 114]. Exposure to CMV-infected fibroblasts drives the generation of this subset *in vitro*, supporting the idea that NK cells expressing NKG2C are preferentially responsive to CMV, likely a result of recognition of a viral antigen in the context of HLA-E, the ligand for NKG2C [109]. There is significant evidence that these NKG2C^+^CD57^+^ NK cells represent a memory-like subset of NK cells (reviewed in [103, 104]). Supporting the idea that these cells are memory-like, NKG2C^+^CD57^+^ NK cells dramatically expand post-transplant in the setting of CMV reactivation, and the subsequent reduction in numbers upon control of viremia—kinetics consistent with a recall response [112, 115–117]. The recent demonstration that these memory-like NK cells have epigenetic changes that are associated with their altered functional capacity provides a critical and important mechanistic explanation for how these memory-like responses might be generated [118–120].

If the existence of NKG2C^+^CD57^+^ NK cells represents a “clonal” expansion of CMV-specific NK cells, then it stands to reason that this clonal expansion would diminish the diversity of the NK cell repertoire and restrict downstream responses. This possibility is raised in the commentary by Achour *et al*., who speculate that CMV infection might decrease NK cell diversity and favor the development of certain tumors [103]. Supporting this idea, NKG2C^+^ NK cells differ in the profile of cytokines produced when compared to immature NK cells from CMV^-^ patients, and in patterns that typically favor tumorigenesis [121–123].

At the surface, the idea that CMV infection narrows NK cell diversity through clonal expansion appears to be in conflict with the data that viral infection and maturity are both associated with increased NK cell repertoire diversity [53, 54, 124]. Yet, delving more deeply into the data, this apparent conflict does not exist. Indeed, the expansion of NKG2C^+^CD57^+^ NK cells can dominate the NK cell repertoire. However, these NK cells still express a vast array of additional activating and inhibitory receptors, and if diversity measures take into account these additional receptors, there is no reduction in NK cell diversity in CMV^+^ individuals [50, 53, 54, 113, 125]. In fact, individuals who were CMV^+^ were not significantly different in their NK cell repertoire diversity than CMV^-^ individuals [50, 54]. Furthermore, we compared the NK diversity between NKG2C^+^ and NKG2C^-^ cells within CMV-seropositive individuals, and found a trend for increased diversity among NKG2C^+^ NK cells (Strauss-Albee and Blish, unpublished). Thus, even with the fixing of expression levels of several receptors due to a clonal-like expansion, the assortment of other activating and inhibitory receptors is sufficient to drive diversification of even these “clonal” cells. While CMV clearly imprints the NK cell repertoire and changes its function, these findings remain consistent with the idea that viral exposure drives NK cell diversification.

## DOES NK CELL DIVERSITY DECREASE THE FLEXIBILITY OF THE NK CELL REPERTOIRE?

**Figure 1.**
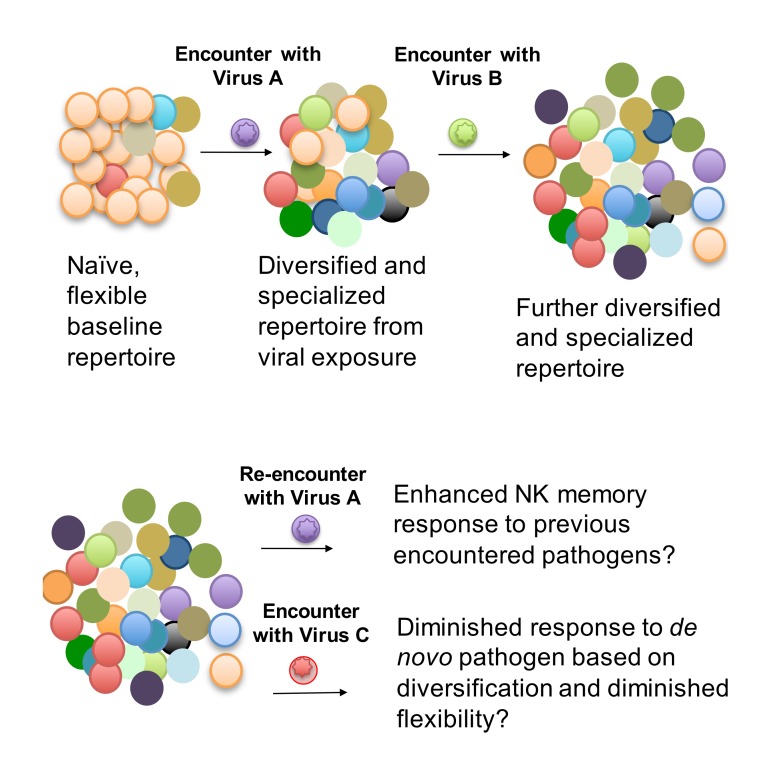
Proposed model of the relationship between NK cell diversity and viral exposure. Based on the association between age, immune experience, and NK cell diversity, I propose that the NK cell repertoire begins as a naïve, flexible repertoire that is relatively homogenous from a phenotypic perspective, though it has extensive diversity in KIR expression patterns based on genetics. Upon encounter with different viruses, the NK cell repertoire diversifies, in part by increasing expression levels of activating receptors, as it seeks to adapt to the viral encounter. Each subsequent encounter further diversifies and specializes the repertoire. This specialization might contribute to memory/recall responses, but may also have the surprising effect of diminishing the ability to respond to de novo pathogens. Vaccine and viral challenge studies in human and animal models will be needed to validate or invalidate this model.

Perhaps the most surprising finding about NK cell diversity in the last several years is the fact that higher pre-infection NK cell diversity is associated with increased risk of HIV acquisition in a small cohort (n = 37) of Kenyan women [54]. At the surface, this is a counterintuitive finding—as immunologists, we always perceive diversity to be a good thing—so why would diversity be associated with increased risk of acquiring HIV? Simply put, the answer is not entirely clear, but there are several possibilities. First, given the difficulty of finding viably preserved peripheral blood mononuclear cells from prior to HIV infection, this was a small study, and may not hold following analysis of additional cohorts. Second, even if true, this is an association, not a causal finding. NK cell diversity might correlate with some other, unmeasured factors that actually are driving the enhanced risk. Despite these caveats, there are some clues as to potential mechanisms that might drive this exposure. Because viral exposure drives NK cell diversity and alters responsiveness [54], the women who acquired HIV infection may have had more HIV exposures, which result in increased NK cell diversity and increased risk. Another intriguing possibility, which needs to be evaluated in future studies, is that serial viral infections may modulate the NK cell repertoire and its responsiveness (Figure 1). According to this proposed model, the NK cell repertoire begins in a relatively homogenous, but very flexible and tunable state. Each viral exposure diversifies and specializes the repertoire, enhancing recall responses for NK memory, but potentially diminishing the response to *de novo* pathogens. Notably, this model of commitment is consistent with data from murine NK cell memory in which memory NK cells, once committed to one pathway, have diminished responses to the other [126]. I propose this model not as proven, but as a framework for future studies and for consideration of the impact of vaccination on NK cell responses. It is critical that we understand the mechanisms by which repeated exposures affect NK cell responses to both on-target and off-target antigens.

## CONCLUSIONS

The advent of new technologies has put us at the cusp of unraveling the answers to many important questions about human NK cells, yet much uncertainty remains. How is NK cell diversity maintained at a stable level, for at least 6 months, when the half-life of NK cells is approximately 2 weeks? Does NK cell diversity truly reflect differentiated NK cells that lack the flexibility to respond to a *de novo* pathogen? Can we identify unique subsets of NK cells that are primed to respond to different viruses or cancers? How does vaccination alter the NK cell repertoire and its responsiveness? In addition to these important questions, it is equally important to consider the limitations of the data presented. The extent to which the phenotype correlates with transcriptional pathways is poorly characterized [63, 127], but might influence future studies, particularly those employing single cell RNA-seq. Finally, I have focused here only on blood NK cells, which are not necessarily representative of the NK cell subsets found in tissues [128–131]. It is an exciting time, and I'm certain that future studies will shed significant light on the dynamics of the NK cell repertoire and its functional responses, and impact on viral susceptibility.

## References

[1] Herberman RB, Nunn ME, Lavrin DH Natural cytotoxic reactivity of mouse lymphoid cells against syngeneic acid allogeneic tumors. I. Distribution of reactivity and specificity. Int J Cancer. 1975;16(2):216–29. PubMed PMID: 50294. 50294 10.1002/ijc.2910160204

[2] Kiessling R, Klein E, Wigzell H “Natural” killer cells in the mouse. I. Cytotoxic cells with specificity for mouse Moloney leukemia cells. Specificity and distribution according to genotype. Eur J Immunol. 1975;5(2):112–7. PubMed PMID: 1234049. doi: 10.1002/eji.1830050208 1234049

[3] Rankin L, Groom J, Mielke LA, Seillet C, Belz GT Diversity, function, and transcriptional regulation of gut innate lymphocytes. Front Immunol. 2013;4:22 PubMed PMID: 23508190. Pubmed Central PMCID: 3600536. doi: 10.3389/fimmu.2013.00022 23508190 PMC3600536

[4] Vanherberghen B, Olofsson PE, Forslund E, Sternberg-Simon M, Khorshidi MA, Pacouret S, Guldevall K, Enqvist M, Malmberg K-J, Mehr R, Onfelt B Classification of human natural killer cells based on migration behavior and cytotoxic response. Blood. 2013;121(8):1326–34. PubMed PMID: 23287857. doi: 10.1182/blood-2012-06-439851 23287857

[5] Imai K, Matsuyama S, Miyake S, Suga K, Nakachi K Natural cytotoxic activity of peripheral-blood lymphocytes and cancer incidence: an 11-year follow-up study of a general population. The Lancet. 2000;356(9244):1795–9. PubMed PMID: 11117911. doi: 10.1016/S0140-6736(00)03231-1 11117911

[6] Borrego F, Larrucea S, Solana R, Tarazona R Editorial: NK Cell-Based Cancer Immunotherapy. Front Immunol. 2016;7:249 PubMed PMID: 27446079. Pubmed Central PMCID: 4921465. doi: 10.3389/fimmu.2016.00249 27446079 PMC4921465

[7] Cheng M, Chen Y, Xiao W, Sun R, Tian Z NK cell-based immunotherapy for malignant diseases. Cell Mol Immunol. 2013;10(3):230–52. PubMed PMID: 23604045. Pubmed Central PMCID: 4076738. doi: 10.1038/cmi.2013.10 23604045 PMC4076738

[8] Berrien-Elliott MM, Romee R, Fehniger TA Improving natural killer cell cancer immunotherapy. Current Opinion in Organ Transplantation. 2015;20(6):671–80. PubMed PMID: 26414502. Pubmed Central PMCID: 4635041. doi: 10.1097/MOT.0000000000000243 26414502 PMC4635041

[9] Rezvani K, Rouce RH The Application of Natural Killer Cell Immunotherapy for the Treatment of Cancer. Front Immunol. 2015;6:578 Pubmed Central PMCID: 4648067. doi: 10.3389/fimmu.2015.00578 26635792 PMC4648067

[10] Biron CA, Byron KS, Sullivan JL Severe herpesvirus infections in an adolescent without natural killer cells. N Engl J Med. 1989 6 29;320(26):1731–5. PubMed PMID: 2543925. doi: 10.1056/NEJM198906293202605 2543925

[11] Eidenschenk C, Dunne J, Jouanguy E, Fourlinnie C, Gineau L, Bacq D, McMahon C, Smith O, Casanova J-L, Abel L, Feighery C A Novel Primary Immunodeficiency with Specific Natural-Killer Cell Deficiency Maps to the Centromeric Region of Chromo-some 8. Am J Hum Genet. 2006;78(4):721–7. PubMed PMID: 16532402. Pubmed Central PMCID: 1424699. doi: 10.1086/503269 16532402 PMC1424699

[12] Tangye SG, Phillips JH, Lanier LL, Nichols KE Cutting Edge: Functional Requirement for SAP in 2B4-Mediated Activation of Human Natural Killer Cells as Revealed by the X-Linked Lymphoproliferative Syndrome. J Immunol. 2000;165(6):2932–6. PubMed PMID: 10975798. 10975798 10.4049/jimmunol.165.6.2932

[13] Nakajima H Patients with X-linked lymphoproliferative disease have a defect in 2B4 receptor-mediated NK cell cytotoxicity. Eur J Immunol. 2000;30(11):3309–18. PubMed PMID: 11093147. doi: 10.1002/1521-4141(200011)30:11<3309:AID-IM-MU3309>3.0.CO;2-3 11093147

[14] Parolini S, Bottino C, Falco M, Augugliaro R, Giliani S, Franceschini R, Ochs HD, Wolf H, Bonnefoy JY, Biassoni R, Moretta L, Notarangelo LD, Moretta A X-linked lymphoproliferative disease. 2B4 molecules displaying inhibitory rather than activating function are responsible for the inability of natural killer cells to kill Epstein-Barr virus-infected cells. J Exp Med. 2000;192(3):337–46. PubMed PMID: 10934222. Pubmed Central PMCID: 2193227 10934222 10.1084/jem.192.3.337 PMC2193227

[15] Bottino C, Falco M, Parolini S, Marcenaro E, Augugliaro R, Sivori S, Landi E, Biassoni R, Notarangelo LD, Moretta L, Moretta A NTB-A [correction of GNTB-A], a novel SH2D1A-associated surface molecule contributing to the inability of natural killer cells to kill Epstein-Barr virus-infected B cells in X-linked lymphoproliferative disease. J Exp Med. 2001;194(3):235–46. PubMed PMID: 11489943. Pubmed Central PMCID: 2193462. 11489943 10.1084/jem.194.3.235 PMC2193462

[16] Li F-Y, Chaigne-Delalande B, Su H, Uzel G, Matthews H, Lenardo MJ XMEN disease: a new primary immunodeficiency affecting Mg2+ regulation of immunity against Epstein-Barr virus. Blood. 2014;123(14):2148–52. PubMed PMID: 24550228. Pubmed Central PMCID: 3975255. doi: 10.1182/blood-2013-11-538686 24550228 PMC3975255

[17] Paust S, Andrian UHV Natural killer cell memory. Nat Immunol. 2011;12(6):500–8. PubMed PMID: 21739673. 21739673 10.1038/ni.2032

[18] Sun JC, Lopez-Verges S, Kim CC, DeRisi JL, Lanier LL NK Cells and Immune “Memory.” J Immunol. 2011 2 2;186(4):1891–7. PubMed PMID: 21289313. Pubmed Central PMCID: 4410097. doi: 10.4049/jimmunol.1003035 21289313 PMC4410097

[19] Cooper MA, Elliott JM, Keyel PA, Yang L, Carrero JA, Yokoyama WM Cytokine-induced memory-like natural killer cells. Proc Natl Acad of Sci USA. 2009;106(6):1915–9. PubMed PMID: 19181844. Pubmed Central PMCID: 2644138. doi: 10.1073/pnas.0813192106 19181844 PMC2644138

[20] Vivier E, Raulet DH, Moretta A, Caligiuri MA, Zitvogel L, Lanier LL, Yokoyama WM, Ugolini S Innate or Adaptive Immunity? The Example of Natural Killer Cells. Science. 2011;331(6013):44–9. PubMed PMID: 21212348. Pubmed Central PMCID: 3089969. doi: 10.1126/science.1198687 21212348 PMC3089969

[21] O Sullivan TE, Sun JC, Lanier LL Natural Killer Cell Memory. Immunity. 2015;43(4):634–45. PubMed PMID: 26488815. Pubmed Central PMCID: 4621966. doi: 10.1016/j.immuni.2015.09.013 26488815 PMC4621966

[22] Manser AR, Weinhold S, Uhrberg M Human KIR repertoires: shaped by genetic diversity and evolution. Immunol Rev. 2015;267(1):178–96. PubMed PMID: 26284478. doi: 10.1111/imr.12316 26284478

[23] Davis MM, Bjorkman PJ T-cell antigen receptor genes and T-cell recognition. Nature. 1988;334(6181):395–402. PubMed PMID: 3043226. doi: 10.1038/334395a0 3043226

[24] Parham P The genetic and evolutionary balances in human NK cell receptor diversity. Semin Immunol. 2008;20(6):311–6. PubMed PMID: 19036608. Pubmed Central PMCID: 3205964. doi: 10.1016/j.smim.2008.10.002 19036608 PMC3205964

[25] Parham P Influence of KIR diversity on human immunity. Adv Exp Med Biol. Boston, MA: Springer US; 2005;560(Chapter 6):47–50. PubMed PMID: 15932019. doi: 10.1007/0-387-24180-9_6 15932019

[26] Anfossi N, André P, Guia S, Falk CS, Roetynck S, Stewart CA, Breso V, Frassati C, Reviron D, Middleton D, Romagné F, Ugolini S, Vivier E Human NK Cell Education by Inhibitory Receptors for MHC Class I. Immunity. 2006;25(2):331–42. PubMed PMID: 16901727. doi: 10.1016/j.immuni.2006.06.013 16901727

[27] Kim S, Poursine-Laurent J, Truscott SM, Lybarger L, Song Y-J, Yang L, French AR, Sunwoo JB, Lemieux S, Hansen TH, Yokoyama WM Licensing of natural killer cells by host major histocompatibility complex class I molecules. Nature. 2005;436(7051):709–13. PubMed PMID: 16079848. doi: 10.1038/nature03847 16079848

[28] Fernandez NC, Treiner E, Vance RE, Jamieson AM, Lemieux S, Raulet DH A subset of natural killer cells achieves self-tolerance without expressing inhibitory receptors specific for self-MHC molecules. Blood. 2005;105(11):4416–23. PubMed PMID: 15728129. Pubmed Central PMCID: 1895026. doi: 10.1182/blood-2004-08-3156 15728129 PMC1895026

[29] Parham P MHC class I molecules and kirs in human history, health and survival. Nat Rev Immunol. 2005;5(3):201–14. PubMed PMID: 15719024. doi: 10.1038/nri1570 15719024

[30] Makrigiannis AP, Parham P The evolution of NK cell diversity. Semin Immunol. 2008;20(6):309–10. PubMed PMID: 18938087. doi: 10.1016/j.smim.2008.09.004 18938087

[31] Goodridge JP, Önfelt B, Malmberg K-J Newtonian cell interactions shape natural killer cell education. Immunol Rev. 2015;267(1):197–213. PubMed PMID: 26284479. Pubmed Central PMCID: 4832384. doi: 10.1111/imr.12325 26284479 PMC4832384

[32] Gendzekhadze K, Norman PJ, Abi-Rached L, Graef T, Moesta AK, Layrisse Z, Parham P Co-evolution of KIR2DL3 with HLA-C in a human population retaining minimal essential diversity of KIR and HLA class I ligands. Proc Natl Acad Sci USA. National Acad Sciences; 2009;106(44):18692–7. PubMed PMID: 19837691. Pubmed Central PMCID: 2774017. doi: 10.1073/pnas.0906051106 19837691 PMC2774017

[33] Hiby SE, Apps R, Sharkey AM, Farrell LE, Gardner L, Mulder A, Claas FH, Walker JJ, Redman CC, Morgan L, Tower C, Regan L, Moore GE, Carrington M, Moffett A Maternal activating KIRs protect against human reproductive failure mediated by fetal HLA-C2. J Clin Invest. 2010;120(11):4102–10. PubMed PMID: 20972337. Pubmed Central PMCID: 2964995. doi: 10.1172/JCI43998 20972337 PMC2964995

[34] Hiby SE, Apps R, Chazara O, Farrell LE, Magnus P, Trogstad L, Gjessing HK, Carrington M, Moffett A Maternal KIR in Combination with Paternal HLA-C2 Regulate Human Birth Weight. J Immunol. 2014;192(11)5069–73. PubMed PMID: 24778445. Pubmed Central PMCID: 4028203. doi: 10.4049/jimmunol.140057 24778445 PMC4028203

[35] Xiong S, Sharkey AM, Kennedy PR, Gardner L, Farrell LE, Chazara O, Bauer J, Hiby SE, Colucci F, Moffett A Maternal uterine NK cell-activating receptor KIR2DS1 enhances placentation. J Clin Invest. 2013;123(10);4264–72. PubMed PMID: 24091323. Pubmed Central PMCID: 4382274. doi: 10.1172/JCI68991 24091323 PMC4382274

[36] Moffett A, Colucci F Co-evolution of NK receptors and HLA ligands in humans is driven by reproduction. Immunol Rev. 2015;267(1):283–97. PubMed PMID: 26284484. doi: 10.1111/imr.12323 26284484

[37] Parham P, Moffett A Variable NK cell receptors and their MHC class I ligands in immunity, reproduction and human evolution. Nat Rev Immunol. Nature Publishing Group; 2013;13(2):133–44. PubMed PMID: 23334245. Pubmed Central PMCID: 3956658. doi: 10.1038/nri3370 23334245 PMC3956658

[38] Hilton HG, Norman PJ, Nemat-Gorgani N, Goyos A, Hollenbach JA, Henn BM, Gignoux CR, Guethlein LA, Parham P Loss and Gain of Natural Killer Cell Receptor Function in an African Hunter-Gatherer Population. Tishkoff SA, editor. PLoS Genet. 2015;11(8):e1005439–20. PubMed PMID: 26292085. Pubmed Central PMCID: 4546388. doi: 10.1371/journal.pgen.1005439 26292085 PMC4546388

[39] Lanier LL, Le AM, Phillips JH, Warner NL, Babcock GF Subpopulations of human natural killer cells defined by expression of the Leu-7 (HNK-1) and Leu-11 (NK-15) antigens. J Immunol. 1983 10;131(4):1789–96. PubMed PMID: 6225799. 6225799

[40] Martinet L, De Andrade LF, Guillerey C, Lee JS, Liu J, Souza-Fonseca-Guimaraes F, Hutchinson DS, Kolesnik TB, Nicholson SE, Huntington ND, Smyth MJ DNAM-1 Expression Marks an Alternative Program of NK Cell Maturation. CellReports. The Authors; 2015;11(1):85–97. PubMed PMID: 25818301. doi: 10.1016/j.celrep.2015.03.006 25818301

[41] Di Santo JP Natural killer cells: diversity in search of a niche. Nat Immunol. 2008;9(5):473–5. PubMed PMID: 18425102. doi: 10.1038/ni.f.201 18425102

[42] Cooper MA, Fehniger TA, Caligiuri MA The biology of human natural killer-cell subsets. Trends Immunol. 2001;22(11):633–40. 2001;22(11):633–40 PubMed PMID: 11698225. 11698225 10.1016/s1471-4906(01)02060-9

[43] Angelo LS, Banerjee PP, Monaco-Shawver L, Rosen JB, Makedonas G, Forbes LR, Mace EM, Orange JS Practical NK cell phenotyping and variability in healthy adults. Immunol Res. 2015;62(3):341–56. PubMed PMID: 26013798. Pubmed Central PMCID: 4470870. doi: 10.1007/s12026-015-8664-y 26013798 PMC4470870

[44] Alter G, Teigen N, Davis BT, Addo MM, Suscovich TJ, Waring MT, Streeck H, Johnston MN, Staller KD, Zaman MT, Yu XG, Lichterfeld M, Basgoz N, Rosenberg ES, Altfeld M Sequential deregulation of NK cell subset distribution and function starting in acute HIV-1 infection. Blood. 2005;106(10):3366–9. PubMed PMID: 16002429. doi: 10.1182/blood-2005-03-1100 16002429

[45] Shilling HG, Young N, Guethlein LA, Cheng NW, Gardiner CM, Tyan D, Parham P Genetic control of human NK cell repertoire. J Immunol. 2002;169(1):239–47. PubMed PMID: 12077250. 12077250 10.4049/jimmunol.169.1.239

[46] Shilling HG, Guethlein LA, Cheng NW, Gardiner CM, Rodriguez R, Tyan D, Parham P Allelic Polymorphism Synergizes with Variable Gene Content to Individualize Human KIR Genotype. J Immunol. 2002;168(5):2307–15. PubMed PMID: 11859120. 11859120 10.4049/jimmunol.168.5.2307

[47] Yawata M Roles for HLA and KIR polymorphisms in natural killer cell repertoire selection and modulation of effector function. J Exp Med. 2006;203(3):633–45. PubMed PMID: 16533882. Pubmed Central PMCID: 2118260. doi: 10.1084/jem.20051884 16533882 PMC2118260

[48] Yawata M, Yawata N, McQueen K, Cheng N, Guethlein L, Rajalingam R, Shilling H, Parham P Predominance of group A KIR haplotypes in Japanese associated with diverse NK cell repertoires of KIR expression. Immunogenetics. 2002;54(8):543–50. PubMed PMID: 12439616. doi: 10.1007/s00251-002-0497-x 12439616

[49] Yawata M, Yawata N, Draghi M, Partheniou F, Little A-M, Parham P MHC class I-specific inhibitory receptors and their ligands structure diverse human NK-cell repertoires toward a balance of missing self-response. Blood. 2008;112(6):2369–80. PubMed PMID: 18583565. Pubmed Central PMCID: 2532809. doi: 10.1182/blood-2008-03-143727 18583565 PMC2532809

[50] Horowitz A, Strauss-Albee DM, Leipold M, Kubo J, Nemat-Gorgani N, Dogan OC, Dekker CL, Mackey S, Maecker H, Swan GE, Davis MM, Norman PJ, Guethlein LA, Desai M, Parham P, Blish CA Genetic and environmental determinants of human NK cell diversity revealed by mass cytometry. Sci Transl Med. 2013;5(208):208ra145 PubMed PMID: 24154599. Pubmed Central PMCID: 3918221. doi: 10.1126/scitranslmed.3006702 PMC3918221 24154599

[51] Bendall SC, Simonds EF, Qiu P, Amir E-AD, Krutzik PO, Finck R, Bruggner RV, Melamed R, Trejo A, Ornatsky OI, Balderas RS, Plevritis SK, Sachs K, Pe'er D, Tanner SD, Nolan GP Single-cell mass cytometry of differential immune and drug responses across a human hematopoietic continuum. Science. 2011;332(6030):687–96. PubMed PMID: 21551058. Pubmed Central PMCID: 3273988. doi: 10.1126/science.1198704 21551058 PMC3273988

[52] Bendall SC, Nolan GP, Roederer M, Chattopadhyay PK A deep profiler's guide to cytometry. Trends Immunol. 2012;33(7):323–32. PubMed PMID: 22476049. Pubmed Central PMCID: 3383392. doi: 10.1016/j.it.2012.02.010 22476049 PMC3383392

[53] Strauss-Albee DM, Horowitz A, Parham P, Blish CA Coordinated regulation of NK receptor expression in the maturing human immune system. J of Immunol. 2014;193(10):4871–9. PubMed PMID: 25288567. Pubmed Central PMCID: 4225175. doi: 10.4049/jimmunol.1401821 25288567 PMC4225175

[54] Strauss-Albee DM, Fukuyama J, Liang EC, Yao Y, Jarrell JA, Drake AL, Kinuthia J, Montgomery RR, John-Stewart G, Holmes S, Blish CA Human NK cell repertoire diversity reflects immune experience and correlates with viral susceptibility. Sci Transl Med. 2015;7(297):297ra115–5. PubMed PMID: 26203083. Pubmed Central PMCID: 4547537. doi: 10.1126/scitranslmed.aac5722 PMC4547537 26203083

[55] Kay AW, Strauss-Albee DM, Blish CA Application of Mass Cytometry (CyTOF) for Functional and Phenotypic Analysis of Natural Killer Cells. Methods Mol Biol. 2016;1441:13–26. PubMed PMID: 27177653. doi: 10.1007/978-1-4939-3684-7_2 27177653 PMC5304457

[56] Manser AR, Uhrberg M Age-related changes in natural killer cell repertoires: impact on NK cell function and immune surveillance. Cancer Immunol Immunother. Springer Berlin Heidelberg; 2015;65(4):417–26. PubMed PMID: 26288343. doi: 10.1007/s00262-015-1750-0 26288343 PMC11028690

[57] Almeida-Oliveira A, Smith-Carvalho M, Porto LC, Cardoso-Oliveira J, Ribeiro ADS, Falcão RR, Abdelhay E, Bouzas LF, Thuler LCS, Ornellas MH, Diamond HR Age-related changes in natural killer cell receptors from childhood through old age. Hum Immunol. 2011;72(4):319–29. PubMed PMID: 21262312. doi: 10.1016/j.humimm.2011.01.009 21262312

[58] Sundström Y, Nilsson C, Lilja G, Karre K, Troye-Blomberg M, Berg L The Expression of Human Natural Killer Cell Receptors in Early Life. Scand J Immunol. 2007;66(2-3):335–44. PubMed PMID: 17635811. doi: 10.1111/j.1365-3083.2007.01980.x 17635811

[59] Le Garff-Tavernier M, Béziat V, Decocq J, Siguret V, Gandjbakhch F, Pautas E, Debré P, Merle-Beral H, Vieillard V Human NK cells display major phenotypic and functional changes over the life span. Aging Cell. 2010;9(4):527–35. PubMed PMID: 20477761. doi: 10.1111/j.1474-9726.2010.00584.x 20477761

[60] Lutz CT, Moore MB, Bradley S, Shelton BJ, Lutgendorf SK Reciprocal age related change in natural killer cell receptors for MHC class I. Mech Ageing Dev. 2005;126(6-7):722–31. PubMed PMID: 15888327. Pubmed Central PMCID: 3394430. doi: 10.1016/j.mad.2005.01.004 15888327 PMC3394430

[61] Wang F, Hou H, Wu S, Tang Q, Liu W, Huang M, Yin B, Huang J, Mao L, Lu Y, Sun Z TIGIT expression levels on human NK cells correlate with functional heterogeneity among healthy individuals. Eur J Immunol. 2015;45(10):2886–97. PubMed PMID: 26171588. doi: 10.1002/eji.201545480 26171588

[62] Hazeldine J, Lord JM The impact of ageing on natural killer cell function and potential consequences for health in older adults. Ageing Res Rev. 2013;12(4):1069–78. PubMed PMID: 23660515. Pubmed Central PMCID: 4147963. doi: 10.1016/j.arr.2013.04.003 23660515 PMC4147963

[63] Goodier MR, White MJ, Darboe A, Nielsen CM, Goncalves A, Bottomley C, Moore SE, Riley EM Rapid NK cell differentiation in a population with near-universal human cytomegalovirus infection is attenuated by NKG2C deletions. Blood. American Society of Hematology; 2014;124(14):2213–22. PubMed PMID: 25150297. Pubmed Central PMCID: 4206953. doi: 10.1182/blood-2014-05-576124 25150297 PMC4206953

[64] Ma Y, Li X, Kuang E Viral Evasion of Natural Killer Cell Activation. Viruses. 2016;8(4):95 PubMed PMID: 27077876. Pubmed Central PMCID: 4848590. doi: 10.3390/v8040095 27077876 PMC4848590

[65] Marras F, Bozzano F, De Maria A Involvement of activating NK cell receptors and their modulation in pathogen immunity. J Biomed Biotechnol. 2011;2011:152430 PubMed PMID: 21860586. Pubmed Central PMCID: 3155793. doi: 10.1155/2011/152430 21860586 PMC3155793

[66] Marras F, Bozzano F, Ascierto ML, De Maria A Baseline and Dynamic Expression of Activating NK Cell Receptors in the Control of Chronic Viral Infections: The Paradigm of HIV-1 and HCV. Front Immunol. 2014;5(6):305 PubMed PMID: 25071766. Pubmed Central PMCID: 4078246. doi: 10.3389/fimmu.2014.00305 25071766 PMC4078246

[67] Zingoni A, Ardolino M, Santoni A, Cerboni C NKG2D and DNAM-1 activating receptors and their ligands in NK-T cell interactions: role in the NK cell-mediated negative regulation of T cell responses. Front Immunol. 2012;3:408 PubMed PMID: 23316196. Pubmed Central PMCID: 3540764. doi: 10.3389/fimmu.2012.00408 23316196 PMC3540764

[68] Reyburn H, Esteso G, Ashiru O, Vales-Gomez M Viral strategies to modulate NKG2D-ligand expression in Human Cytomegalovirus infection. New Horizons in Translational Medicine. Elsevier; 2015;2(6-7):159–66. doi: 10.1016/j.nhtm.2015.11.002

[69] Carrillo-Bustamante P, Kesmir C, De Boer RJ The evolution of natural killer cell receptors. Immunogenetics. 2016;68(1):3–18. PubMed PMID: 26392015. Pubmed Central PMCID: 4701786. doi: 10.1007/s00251-015-0869-7 26392015 PMC4701786

[70] Cheent K, Khakoo SI Natural killer cells: integrating diversity with function. Immunology. 2009;126(4):449–57. PubMed PMID: 19278418. Pubmed Central PMCID: 2673357. doi: 10.1111/j.1365-2567.2009.03045.x 19278418 PMC2673357

[71] Körner C, Altfeld M Role of KIR3DS1 in human diseases. Front Immunol. 2012;3:326 PubMed PMID: 23125843. Pubmed Central PMCID: 3485674. doi: 10.3389/fimmu.2012.00326 23125843 PMC3485674

[72] Martin MP, Gao X, Lee J-H, Nelson GW, Detels R, Goedert JJ, Buchbinder S, Hoots K, Vlahov D, Trowsdale J, Wilson M, O'Brien SJ, Carrington M Epistatic interaction between KIR3DS1 and HLA-B delays the progression to AIDS. Nat Genet. 2002;31(4):429–34. PubMed PMID: 12134147. doi: 10.1038/ng934 12134147

[73] Martin MP, Qi Y, Gao X, Yamada E, Martin JN, Pereyra F, Colombo S, Brown EE, Shupert WL, Phair J, Goedert JJ, Buchbinder S, Kirk GD, Telenti A, Connors M, O'Brien SJ, Walker BD, Parham P, Deeks SG, Mcvicar DW, Carrington M Innate partnership of HLA-B and KIR3DL1 subtypes against HIV-1. Nat Genet. 2007;39(6):733–40. PubMed PMID: 17496894. Pubmed Central PMCID: 4135476. doi: 10.1038/ng2035 17496894 PMC4135476

[74] López-Vázquez A, Miña-Blanco A, Martínez-Borra J, Njobvu PD, Suárez-Alvarez B, Blanco-Gelaz MA, González S, Rodrigo L, López-Larrea C Interaction between KIR3DL1 and HLA-B*57 supertype alleles influences the progression of HIV-1 infection in a Zambian population. HIM. 2005;66(3):285–9. PubMed PMID: 15784466. doi: 10.1016/j.humimm.2005.01.001 15784466

[75] Pelak K, Need AC, Fellay J, Shianna KV, Feng S, Urban TJ, Ge D, De Luca A, Martinez-Picado J, Wolinsky SM, Martinson JJ, Jamieson BD, Bream JH, Martin MP, Borrow P, Letvin NL, Mcmichael AJ, Haynes BF, Telenti A, Carrington M, Goldstein DB, Alter G Copy Number Variation of KIR Genes Influences HIV-1 Control. PLoS Biol. 2011;9(11):e1001208 PubMed PMID: 22140359. Pubmed Central PMCID: 3226550. doi: 10.1371/journal.pbio.1001208 22140359 PMC3226550

[76] Jennes W, Verheyden S, Mertens JW, Camara M, Seydi M, Dieye TN, Mboup S, Demanet C, Kestens L Inhibitory KIR/HLA ligand incompatibility between sexual partners confers protection against HIV-1 transmission. Blood. 2013;121(7):1157–64. PubMed PMID: 23243280. doi: 10.1182/blood-2012-09-455352 23243280

[77] Alter G, Rihn S, Walter K, Nolting A, Martin M, Rosenberg ES, Miller JS, Carrington M, Altfeld M HLA Class I Subtype-Dependent Expansion of KIR3DS1+ and KIR3DL1+ NK Cells during Acute Human Immunodeficiency Virus Type 1 Infection. J Virol. 2009;83(13):6798–805. PubMed PMID: 19386717. Pubmed Central PMCID: 2698561. doi: 10.1128/JVI.00256-09 19386717 PMC2698561

[78] Alter G, Martin MP, Teigen N, Carr WH, Suscovich TJ, Schneidewind A, Streeck H, Waring M, Meier A, Brander C, Lifson JD, Allen TM, Carrington M, Altfeld M Differential natural killer cell-mediated inhibition of HIV-1 replication based on distinct KIR/HLA subtypes. J Exp Med. 2007;204(12):3027–36. PubMed PMID: 19386717. Pubmed Central PMCID: 2698561. doi: 10.1128/JVI.00256-09 18025129 PMC2118524

[79] Song R, Lisovsky I, Lebouché B, Routy J-P, Bruneau J, Bernard NF HIV Protective KIR3DL1/S1-HLA-B Genotypes Influence NK Cell-Mediated Inhibition of HIV Replication in Autologous CD4 Targets. PLoS Pathog. 2014;10(1):e1003867–12. PubMed PMID: 24453969. Pubmed Central PMCID: 3894215. doi: 10.1371/journal.ppat.1003867 24453969 PMC3894215

[80] Carr WH, Rosen DB, Arase H, Nixon DF, Michaëlsson J, Lanier LL Cutting Edge: KIR3DS1, a gene implicated in resistance to progression to AIDS, encodes a DAP12-associated receptor expressed on NK cells that triggers NK cell activation. J Immunol. 2007;178(2):647–51. PubMed PMID: 17202323. Pubmed Central PMCID: 2561215. 17202323 10.4049/jimmunol.178.2.647 PMC2561215

[81] Norman PJ, Abi-Rached L, Gendzekhadze K, Korbel D, Gleimer M, Rowley D, Bruno D, Carrington CVF, Chandanayingyong D, Chang Y-H, Crespí C, Saruhan-Direskeneli G, Fraser PA, Hameed K, Kamkamidze G, Koram KA, Layrisse Z, Matamoros N, Milà J, Park MH, Pitchappan RM, Ramdath DD, Shiau M-Y, Stephens HAF, Struik S, Verity DH, Vaughan RW, Tyan D, Davis RW, Riley EM, Ronaghi M, Parham P Unusual selection on the KIR3DL1/S1 natural killer cell receptor in Africans. Nat Genet. 2007;39(9):1092–9. PubMed PMID: 17694054. doi: 10.1038/ng2111 17694054

[82] Cella M, Longo A, Ferrara GB, Strominger JL, Colonna M NK3-specific natural killer cells are selectively inhibited by Bw4-positive HLA alleles with isoleucine 80. J Exp Med. 1994;180(4):1235–42. PubMed PMID: 7931060. Pubmed Central PMCID: 2191670. 7931060 10.1084/jem.180.4.1235 PMC2191670

[83] Gillespie GMA, Bashirova A, Dong T, Mcvicar DW, Rowland-Jones SL, Carrington M Lack of KIR3DS1 binding to MHC class I Bw4 tetramers in complex with CD8+ T cell epitopes. AIDS Res Hum Retroviruses. 2007;23(3):451–5. PubMed PMID: 17411378. doi: 10.1089/aid.2006.0165 17411378

[84] O'Connor GMG, Guinan KJK, Cunningham RTR, Middleton DD, Parham PP, Gar-diner CMC Functional polymorphism of the KIR3DL1/S1 receptor on human NK cells. 2007;178(1):235–41. PubMed PMID: 17182560. 10.4049/jimmunol.178.1.235 17182560

[85] O'Connor GM, Vivian JP, Gostick E, Pymm P, Lafont BAP, Price DA, Rossjohn J, Brooks AG, Mcvicar DW Peptide-Dependent Recognition of HLA-B*57:01 by KIR3DS1. J Virol. 2015;89(10):5213–21. PubMed PMID: 25740999. Pubmed Central PMCID: 4442525. doi: 10.1128/JVI.03586-14 25740999 PMC4442525

[86] Garcia-Beltran WF, Hölzemer A, Martrus G, Chung AW, Pacheco Y, Simoneau CR, Rucevic M, Lamothe-Molina PA, Pertel T, Kim T-E, Dugan H, Alter G, Déchanet-Merville J, Jost S, Carrington M, Altfeld M Open conformers of HLA-F are high-affinity ligands of the activating NK-cell receptor KIR3DS1. Nat Immunol. 2016;17(9):1067–74. PubMed PMID: 27455421. Pubmed Central PMCID: 4992421. doi: 10.1038/ni.3513. 27455421 PMC4992421

[87] Boudreau JE, Mulrooney TJ, Le Luduec J-B, Barker E, Hsu KC KIR3DL1 and HLA-B Density and Binding Calibrate NK Education and Response to HIV. J Immunol. 2016;196(8):3398–410. PubMed PMID: 26962229. Pubmed Central PMCID: 4868784. doi: 10.4049/jimmunol.1502469 26962229 PMC4868784

[88] Hens J, Jennes W, Kestens L The role of NK cells in HIV-1 protection: autologous, allogeneic or both? AIDS Res Ther. 2016;13(1):15 PubMed PMID: 26997965. Pubmed Central PMCID: 4799629. doi: 10.1186/s12981-016-0099-6 26997965 PMC4799629

[89] Lisovsky I, Isitman G, Song R, DaFonseca S, Tremblay-McLean A, Lebouché B, Routy J-P, Bruneau J, Bernard NF A Higher Frequency of NKG2A+ than of NKG2A-NK Cells Responds to Autologous HIV-Infected CD4 Cells irrespective of Whether or Not They Coexpress KIR3DL1. J Virol. 2015;89(19):9909–19. PubMed PMID: 26202228. Pubmed Central PMCID: 4577891. doi: 10.1128/JVI.01546-15 26202228 PMC4577891

[90] Davis ZB, Cogswell A, Scott H, Mertsching A, Boucau J, Wambua D, Le Gall S, Planelles V, Campbell KS, Barker E A Conserved HIV-1-Derived Peptide Presented by HLA-E Renders Infected T-cells Highly Susceptible to Attack by NKG2A/CD94-Bearing Natural Killer Cells. PLoS Pathog. 2016;12(2):e1005421–2. PubMed PMID: 26828202. Pubmed Central PMCID: 4735451. doi: 10.1371/journal.ppat.1005421 26828202 PMC4735451

[91] Lisovsky I, Isitman G, Tremblay-McLean A, Song R, DaFonnseca S, Lebouché B, Routy J-P, Bruneau J, Bernard NF The differential impact of NK cell education via KIR2DL3 and KIR3DL1 on CCL4 secretion in the context of in vitro HIV infection. Clin Exp Immunol. 2016;1–42. PubMed PMID: 27506421. doi: 10.1111/cei.12849 PMC5108064 27506421

[92] Ahlenstiel G, Martin MP, Gao X, Carrington M, Rehermann B Distinct KIR/HLA compound genotypes affect the kinetics of human antiviral natural killer cell responses. J Clin Invest. 2008:1–10. PubMed PMID: 18246204. Pubmed Central PMCID: 2214845. doi: 10.1172/JCI32400 PMC2214845 18246204

[93] La D, Czarnecki C, El-Gabalawy H, Kumar A, Meyers AFA, Bastien N, Simonsen JN, Plummer FA, Luo M Enrichment of Variations in KIR3DL1/S1 and KIR2DL2/L3 among H1N1/09 ICU Patients: An Exploratory Study. PLoS ONE. 2011;6(12):e29200 PubMed PMID: 22216211. Pubmed Central PMCID: 3247251. doi: 10.1371/journal.pone.0029200 22216211 PMC3247251

[94] Mendelson M, Tekoah Y, Zilka A, Gershoni-Yahalom O, Gazit R, Achdout H, Bovin NV, Meningher T, Mandelboim M, Mandelboim O, David A, Porgador A NKp46 O-Glycan Sequences That Are Involved in the Interaction with Hemagglutinin Type 1 of Influenza Virus. J Virol. 2010;84(8):3789–97. PubMed PMID: 20147410. Pubmed Central PMCID: 2849520. doi: 10.1128/JVI.01815-09 20147410 PMC2849520

[95] Draghi M, Pashine A, Sanjanwala B, Gendzekhadze K, Cantoni C, Cosman D, Moretta A, Valiante NM, Parham P NKp46 and NKG2D recognition of infected dendritic cells is necessary for NK cell activation in the human response to influenza infection. 2007;178(5):2688–98. PubMed PMID: 17312110. 10.4049/jimmunol.178.5.2688 17312110

[96] Bar-On Y, Glasner A, Meningher T, Achdout H, Gur C, Lankry D, Vitenshtein A, Meyers AFA, Mandelboim M, Mandelboim O Neuraminidase-Mediated, NKp46-Dependent Immune-Evasion Mechanism of Influenza Viruses. Cell Rep. 2013;3(4):1044–50. PubMed PMID: 23602571. Pubmed Central PMCID: 3863986. doi: 10.1016/j.celrep.2013.03.034 23602571 PMC3863986

[97] Narni-Mancinelli E, Jaeger BN, Bernat C, Fenis A, Kung S, De Gassart A, Mahmood S, Gut M, Heath SC, Estellé J, Bertosio E, Vély F, Gastinel LN, Beutler B, Malissen B, Malissen M, Gut IG, Vivier E, Ugolini S Tuning of natural killer cell reactivity by NKp46 and Helios calibrates T cell responses. Science. 2012;335(6066):344–8. 2012;335(6066):344-8 PubMed PMID: 22267813. doi: 10.1126/science.1215621 22267813

[98] Duev-Cohen A, Bar-On Y, Glasner A, Berhani O, Ophir Y, Levi-Schaffer F, Mandelboim M, Mandelboim O The human 2B4 and NTB-A receptors bind the influenza viral hemagglutinin and co-stimulate NK cell cytotoxicity. Oncotarget. 2016;7(11):13093–105. PubMed PMID: 26919106. Pubmed Central PMCID: 4914344. doi: 10.18632/oncotarget.7597 26919106 PMC4914344

[99] Amadei B, Urbani S, Cazaly A, Fisicaro P, Zerbini A, Ahmed P, Missale G, Ferrari C, Khakoo SI Activation of natural killer cells during acute infection with hepatitis C virus. Gastroenterology. 2010;138(4):1536–45. PubMed PMID: 20080094. Pubmed Central PMCID: 4183834. doi: 10.1053/j.gastro.2010.01.006 20080094 PMC4183834

[100] Khakoo SI, Thio CL, Martin MP, Brooks CR, Gao X, Astemborski J, Cheng J, Goedert JJ, Vlahov D, Hilgartner M, Cox S, Little A-M, Alexander GJ, Cramp ME, O'Brien SJ, Rosenberg WMC, Thomas DL, Carrington M HLA and NK cell inhibitory receptor genes in resolving hepatitis C virus infection. Science. 2004;305(5685):872–4. PubMed PMID: 15297676. doi: 10.1126/science.1097670 15297676

[101] Thoens C, Berger C, Trippler M, Siemann H, Lutterbeck M, Broering R, Schlaak J, Heinemann FM, Heinold A, Nattermann J, Scherbaum N, Alter G, Timm J KIR2DL3⊠NKG2A⊠ natural killer cells are associated with protection from productive hepatitis C virus infection in people who inject drugs. J Hepatol. 2014 9;61(3):475–81. PubMed PMID: 24780303. doi: 10.1016/j.jhep.2014.04.020 24780303

[102] Carapito R, Bahram S Genetics, genomics, and evolutionary biology of NKG2D ligands. Immunol Rev. 2015;267(1):88–116. PubMed PMID: 26284473. doi: 10.1111/imr.12328 26284473

[103] Achour A, Baychelier F, Marty M, Debré P, Samuel D, Vieillard V Transplantation-induced cancers: Emerging evidence that clonal CMV-specific NK cells are causal immunogenic factors. Oncoimmunology. 2014;3(5):e28782–3. PubMed PMID: 25050225. Pubmed Central PMCID: 4077860. doi: 10.4161/onci.28782 25050225 PMC4077860

[104] Rölle A, Brodin P Immune Adaptation to Environmental Influence: The Case of NK Cells and HCMV. Trends Immunol. Elsevier; 2016;37(3):233–43. PubMed PMID: 26869205. doi: 10.1016/j.it.2016.01.005 26869205

[105] Chiesa Della M Impact of HCMV infection on NK cell development and function after HSCT. Front Immunol. 2013;4:458 PubMed PMID: 24379818. Pubmed Central PMCID: 3861788. doi: 10.3389/fimmu.2013.00458 24379818 PMC3861788

[106] Gumá MM, Cabrera CC, Erkizia II, Bofill MM, Clotet BB, Ruiz LL, López-Botet MM Human cytomegalovirus infection is associated with increased proportions of NK cells that express the CD94/NKG2C receptor in aviremic HIV-1-positive patients. J Infect Dis. 2006;194(1):38–41. PubMed PMID: 16741880. doi: 10.1086/504719 16741880

[107] Djaoud Z, David G, Bressollette C, Willem C, Rettman P, Gagne K, Legrand N, Mehlal S, Cesbron A, Imbert-Marcille BM, Retiere C Amplified NKG2C+ NK Cells in Cytomegalovirus (CMV) Infection Preferentially Express Killer Cell Ig-like Receptor 2DL: Functional Impact in Controlling CMV-Infected Dendritic Cells. J Immunol. 2013;191(5):2708–16. PubMed PMID: 23918974. doi: 10.4049/jimmunol.1301138 23918974

[108] Guma M Imprint of human cytomegalovirus infection on the NK cell receptor repertoire. Blood. 2004;104(12):3664–71. PubMed PMID: 15304389. doi: 10.1182/blood-2004-05-2058 15304389

[109] Gumá M, Budt M, Sáez A, Brckalo T, Hengel H, Angulo A, López-Botet M Expansion of CD94/NKG2C+ NK cells in response to human cytomegalovirus-infected fibroblasts. Blood. 2006;107(9):3624–31. PubMed PMID: 16384928. doi: 10.1182/blood-2005-09-3682 16384928

[110] Lopez-Verges S, Milush JM, Schwartz BS, Pando MJ, Jarjoura J, York VA, Houchins JP, Miller S, Kang S-M, Norris PJ, Nixon DF, Lanier LL Expansion of a unique CD57+NKG2Chi natural killer cell subset during acute human cytomegalovirus infection. 2011 pp. 14725–32. PubMed PMID: 21825173. Pubmed Central PMCID: 3169160. doi: 10.1073/pnas.1110900108 PMC3169160 21825173

[111] Hendricks DW, Balfour HH, Dunmire SK, Schmeling DO, Hogquist KA, Lanier LL Cutting Edge: NKG2ChiCD57+ NK Cells Respond Specifically to Acute Infection with Cytomegalovirus and Not Epstein-Barr Virus. J Immunol. 2014;192(10):4492–6. PubMed PMID: 24740502. Pubmed Central PMCID: 4013527. doi: 10.4049/jimmunol.1303211 24740502 PMC4013527

[112] Foley B, Cooley S, Verneris MR, Pitt M, Curtsinger J, Luo X, Lopez-Verges S, Lanier LL, Weisdorf D, Miller JS Cytomegalovirus reactivation after allogeneic transplantation promotes a lasting increase in educated NKG2C+ natural killer cells with potent function. Blood. 2012;119(11):2665–74. PubMed PMID: 22180440. Pubmed Central PMCID: 3311280. doi: 10.1182/blood-2011-10-386995 22180440 PMC3311280

[113] Béziat V, Dalgard O, Asselah T, Halfon P, Bedossa P, Boudifa A, Hervier B, Theodorou I, Martinot M, Debré P, Björkström NK, Malmberg K-J, Marcellin P, Vieillard V CMV drives clonal expansion of NKG2C+ NK cells expressing self-specific KIRs in chronic hepatitis patients. Eur J Immunol. 2011;42(2):447–57. PubMed PMID: 22105371. doi: 10.1002/eji.201141826 22105371

[114] Beziat V, Liu L, Malmberg JA, Ivarsson MA, Sohlberg E, Bjorklund AT, Retiere C, Sverremark-Ekstrom E, Traherne J, Ljungman P, Schaffer M, Price DA, Trowsdale J, Michaelsson J, Ljunggren HG, Malmberg K-J NK cell responses to cytomegalovirus infection lead to stable imprints in the human KIR repertoire and involve activating KIRs. Blood. 2013;121(14):2678–88. PubMed PMID: 23325834. Pubmed Central PMCID: 3617633. doi: 10.1182/blood-2012-10-459545 23325834 PMC3617633

[115] Achour A, Baychelier F, Besson C, Arnoux A, Marty M, Hannoun L, Samuel D, Debre P, Vieillard V, the K-GREF Study Group. Expansion of CMV-Mediated NKG2C+ NK Cells Associates with the Development of Specific De Novo Malignancies in Liver-Transplanted Patients. J Immunol. 2014;192(1):503–11. PubMed PMID: 24307732. doi: 10.4049/jimmunol.1301951 24307732

[116] Della Chiesa M, Falco M, Podesta M, Locatelli F, Moretta L, Frassoni F, Moretta A Phenotypic and functional heterogeneity of human NK cells developing after umbilical cord blood transplantation: a role for human cytomegalovirus? Blood. 2012;119(2):399–410. PubMed PMID: 22096237. doi: 10.1182/blood-2011-08-372003 22096237

[117] Muñoz-Cobo B, Solano C, Benet I, Costa E, Remigia MJ, la Cámara de R, Nieto J, López J, Amat P, Garcia-Noblejas A, Bravo D, Clari MÁ, Navarro D Functional profile of cytomegalovirus (CMV)-specific CD8+ T cells and kinetics of NKG2C+ NK cells associated with the resolution of CMV DNAemia in allogeneic stem cell transplant recipients. J Med Virol. 2012;84(2):259–67. PubMed PMID: 22170546. doi: 10.1002/jmv.22254 22170546

[118] Zhang T, Scott JM, Hwang I, Kim S Cutting edge: antibody-dependent memory-like NK cells distinguished by FcRγ deficiency. J Immunol. 2013 2 15;190(4):1402–6. PubMed PMID: 23345329. Pubmed Central PMCID: 3623944. doi: 10.4049/jimmunol.1203034 23345329 PMC3623944

[119] Lee J, Zhang T, Hwang I, Kim A, Nitschke L, Kim M, Scott JM, Kamimura Y, Lanier LL, Kim S Epigenetic modification and antibody-dependent expansion of memory-like NK cells in human cytomegalovirus-infected individuals. Immunity. 2015;42(3):431–42. Pubmed Central PMCID: 4537797. doi: 10.1016/j.immuni.2015.02.013 25786175 PMC4537797

[120] Schlums H, Cichocki F, Tesi B, Theorell J, Béziat V, Holmes TD, Han H, Chiang SCC, Foley B, Mattsson K, Larsson S, Schaffer M, Malmberg K-J, Ljunggren H-G, Miller JS, Bryceson YT Cytomegalovirus Infection Drives Adaptive Epigenetic Diversification of NK Cells with Altered Signaling and Effector Function. Immunity. 2015;42(3):443–56. PubMed PMID: 25786176. Pubmed Central PMCID: 4612277. doi: 10.1016/j.immuni.2015.02.008 25786176 PMC4612277

[121] Achour A, Baychelier F, Besson C, Arnoux A, Marty M, Hannoun L, Samuel D, Debré P, Vieillard V, K-GREF Study Group. Expansion of CMV-mediated NKG2C+ NK cells associates with the development of specific de novo malignancies in liver-transplanted patients. J of Immunol. 2014 1 1;192(1):503–11. PubMed PMID: 24307732. doi: 10.4049/jimmunol.1301951 24307732

[122] Ménard C, Blay J-Y, Borg C, Michiels S, Ghiringhelli F, Robert C, Nonn C, Chaput N, Taïeb J, Delahaye NF, Flament C, Emile J-F, Le Cesne A, Zitvogel L Natural killer cell IFN-gamma levels predict long-term survival with imatinib mesylate therapy in gastrointestinal stromal tumor-bearing patients. Cancer Res. 2009;69(8):3563–9. PubMed PMID: 19351841. doi: 10.1158/0008-5472.CAN-08-3807 19351841

[123] Di Cocco P, Soker T, Clemente K, Margiotta G, Coletti G, Lombardi L, Orlando G, Famulari A, Pisani F Cytomegalovirus and gastric cancer after renal transplantation: a possible interplay. Transplant Proc. 2012;44(7):1912–5. PubMed PMID: 22974869. doi: 10.1016/j.transproceed.2012.06.051 22974869

[124] Strauss-Albee DM, Blish CA Human NK Cell Diversity in Viral Infection: Ramifications of Ramification. Front Immunol. 2016;7:66 PubMed PMID: 26973646. Pubmed Central PMCID: 4776076. doi: 10.3389/fimmu.2016.00066 26973646 PMC4776076

[125] Béziat V, Traherne J, Malmberg J-A, Ivarsson MA, Björkström NK, Retière C, Ljunggren H-G, Michaëlsson J, Trowsdale J, Malmberg K-J Tracing dynamic expansion of human NK-cell subsets by high-resolution analysis of KIR repertoires and cellular differentiation. Eur J Immunol. 2014;44(7):2192–6. PubMed PMID: 24723455. Pubmed Central PMCID: 4282447. doi: 10.1002/eji.201444464 24723455 PMC4282447

[126] Min-Oo G, Lanier LL Cytomegalovirus generates long-lived antigen-specific NK cells with diminished bystander activation to heterologous infection. J Exp Med. 2014;211(13):2669–80. PubMed PMID: 25422494. Pubmed Central PMCID: 4267234. doi: 10.1084/jem.20141172 25422494 PMC4267234

[127] Bezman NA, Kim CC, Sun JC, Min-Oo G, Hendricks DW, Kamimura Y, Best JA, Goldrath AW, Lanier LL, Immunological Genome Project C. Molecular definition of the identity and activation of natural killer cells. Nat Immunol. 2012;13(10):1000–9. PubMed PMID: 22902830. Pubmed Central PMCID: 3572860. doi: 10.1038/ni.2395 22902830 PMC3572860

[128] Björkström NK, Ljunggren H-G, Michaëlsson J Emerging insights into natural killer cells in human peripheral tissues. Nat Rev Immunol. 2016;16(5):310–20. PubMed PMID: 27121652.10.1038/nri.2016.34 27121652 10.1038/nri.2016.34

[129] Sojka DK, Tian Z, Yokoyama WM Tissue-resident natural killer cells and their potential diversity. Semin Immunol. 2014;26(2):127–31. PubMed PMID: 24548893. Pubmed Central PMCID: 4459495. doi: 10.1016/j.smim.2014.01.010 24548893 PMC4459495

[130] Tang L, Peng H, Zhou J, Chen Y, Wei H, Sun R, Yokoyama WM, Tian Z Differential phenotypic and functional properties of liver-resident NK cells and mucosal ILC1s. J Autoimmun. 2016;67:29–35. PubMed PMID: 26422992. doi: 10.1016/j.jaut.2015.09.004 26422992

[131] Ivanova D, Krempels R, Ryfe J, Weitzman K, Stephenson D, Gigley JP Review Article NK Cells in Mucosal Defense against Infection. Ivanova D, Krempels R, Ryfe J, Weitzman K, Stephenson D, Gigley JP NK cells in mucosal defense against infection. Biomed Res Int. 2014;2014:413982 PubMed PMID: 25197644. Pubmed Central PMCID: 4150440. doi: 10.1155/2014/413982 25197644 PMC4150440

[132] Foley B, Cooley S, Verneris MR, Curtsinger J, Luo X, Waller EK, Anasetti C, Weisdorf D, Miller JS Human cytomegalovirus (CMV)-induced memory-like NKG2C(+) NK cells are transplantable and expand in vivo in response to recipient CMV antigen. J Immunol. 2012;189(10):5082–8. PubMed PMID: 23077239. Pubmed Central PMCID: 3490031. doi: 10.4049/jimmunol.1201964 23077239 PMC3490031

[133] Muntasell A, López-Montañés M, Vera A, Heredia G, Romo N, Peñafiel J, Moraru M, Vila J, Vilches C, López-Botet M NKG2Czygosity influences CD94/NKG2C receptor function and the NK-cell compartment redistribution in response to human cytomegalovirus. Eur J Immunol. 2013;43(12):3268–78. PubMed PMID: 24030638. doi: 10.1002/eji.201343773 24030638

[134] Prod'homme V, Griffin C, Aicheler RJ, Wang ECY, McSharry BP, Rickards CR, Stanton RJ, Borysiewicz LK, López-Botet M, Wilkinson GWG, Tomasec P The human cytomegalovirus MHC class I homolog UL18 inhibits LIR-1+ but activates LIR-1-NK cells. J Immunol. 2007;178(7):4473–81. PubMed PMID: 17372005. Pubmed Central PMCID: 2843079. 17372005 10.4049/jimmunol.178.7.4473 PMC2843079

[135] Arnon TI, Achdout H, Levi O, Markel G, Saleh N, Katz G, Gazit R, Gonen-Gross T, Hanna J, Nahari E, Porgador A, Honigman A, Plachter B, Mevorach D, Wolf DG, Mandelboim O Inhibition of the NKp30 activating receptor by pp65 of human cytomegalovirus. Nat Immunol. 2005;6(5):515–23. PubMed PMID: 15821739. doi: 10.1038/ni1190 15821739

[136] Martini F, Agrati C, D'Offizi G, Poccia F HLA-E up-regulation induced by HIV infection may directly contribute to CD94-mediated impairment of NK cells. Int J Immunopathol Pharmacol. 2005;18(2):269–76. PubMed PMID: 15888249. 15888249 10.1177/039463200501800209

[137] Zhou J, Amran FS, Kramski M, Angelovich TA, Elliott J, Hearps AC, Price P, Jaworowski A An NK Cell Population Lacking FcRgamma Is Expanded in Chronically Infected HIV Patients. J Immunol. 2015;194(10):4688–97. PubMed PMID: 25855354. doi: 10.4049/jimmunol.1402448 25855354

[138] Merino AM, Dugast A-S, Wilson CM, Goepfert PA, Alter G, Kaslow RA, Tang J KIR2DS4 Promotes HIV-1 Pathogenesis: New Evidence from Analyses of Immunogenetic Data and Natural Killer Cell Function. Unutmaz D, editor. PLoS ONE. 2014;9(6):e99353 PubMed PMID: 24901871. Pubmed Central PMCID: 4047121. doi: 10.1371/journal.pone.0099353 24901871 PMC4047121

[139] Jiang Y, Chen O, Cui C, Zhao B, Han X, Zhang Z, Liu J, Xu J, Hu Q, Liao C, Shang H KIR3DS1/L1 and HLA-Bw4-80I are associated with HIV disease progression among HIV typical progressors and long-term nonprogressors. BMC Infect Dis. BioMed Central; 2013;13(1):405 PubMed PMID: 24059286. Pubmed Central PMCID: 3766012. doi: 10.1186/1471-2334-13-405 24059286 PMC3766012

[140] Boulet S, Song R, Kamya P, Bruneau J, Shoukry NH, Tsoukas CM, Bernard NF HIV protective KIR3DL1 and HLA-B genotypes influence NK cell function following stimulation with HLA-devoid cells. J Immunol. 2010;184(4):2057–64. PubMed PMID: 20061407. doi: 10.4049/jimmunol.0902621 20061407

[141] Parsons MS, Wren L, Isitman G, Navis M, Stratov I, Bernard NF, Kent SJ HIV infection abrogates the functional advantage of natural killer cells educated through KIR3DL1/HLA-Bw4 interactions to mediate anti-HIV antibody-dependent cellular cytotoxicity. J Virol. 2012;86(8):4488–95. PubMed PMID: 22345455. Pubmed Central PMCID: 3318670. doi: 10.1128/JVI.06112-11 22345455 PMC3318670

[142] Alter G, Heckerman D, Schneidewind A, Fadda L, Kadie CM, Carlson JM, Oniangue-Ndza C, Martin M, Li B, Khakoo SI, Carrington M, Allen TM, Altfeld M HIV-1 adaptation to NK-cell-mediated immune pressure. Nature. 2011;476(7358):96–100. PubMed PMID: 21814282. Pubmed Central PMCID: 3194000. doi: 10.1038/nature10237 21814282 PMC3194000

[143] Körner C, Granoff ME, Amero MA, Sirignano MN, Vaidya SA, Jost S, Allen TM, Rosenberg ES, Altfeld M Increased frequency and function of KIR2DL1-3 +NK cells in primary HIV-1 infection are determined by HLA-Cgroup haplotypes. Eur J Immunol. 2014;44(10):2938–48. PubMed PMID: 25043727. Pubmed Central PMCID: 4197106. doi: 10.1002/eji.201444751 25043727 PMC4197106

[144] Altfeld M, Gale M Jr Innate immunity against HIV-1 infection. Nat Immunol. 2015;16(6):554–62. PubMed PMID: 25988887. doi: 10.1038/ni.3157 25988887

[145] Chavan VR, Ahir S, Ansari Z, Samant-Mawani P, Nanavati R, Mehta P, Mania-Pramanik J Diversity in KIR gene repertoire in HIV-1 exposed infected and uninfected infants: A study from India. J Med Virol. 2015;88(3):417–25. PubMed PMID: 26255774. doi: 10.1002/jmv.24348 26255774

[146] Hölzemer A, Thobakgale CF, Jimenez Cruz CA, Garcia-Beltran WF, Carlson JM, van Teijlingen NH, Mann JK, Jaggernath M, Kang S-G, Körner C, Chung AW, Schafer JL, Evans DT, Alter G, Walker BD, Goulder PJ, Carrington M, Hartmann P, Pertel T, Zhou R, Ndung'u T, Altfeld M Selection of an HLA-C*03:04-Restricted HIV-1 p24 Gag Sequence Variant Is Associated with Viral Escape from KIR2DL3+ Natural Killer Cells: Data from an Observational Cohort in South Africa. PLoS Med. 2015;12(11):e1001900; discussion e PubMed PMID: 26575988. Pubmed Central PMCID: 4648589. doi: 10.1371/journal.pmed.1001900 26575988 PMC4648589

[147] Scott-Algara D, Arnold V, Didier C, Kattan T, Pirozzi G, Barré-Sinoussi F, Pancino G The CD85j+ NK cell subset potently controls HIV-1 replication in autologous dendritic cells. PLoS One. 2008;3(4):e1975 PubMed PMID: 18398485. Pubmed Central PMCID: 2276866. doi: 10.1371/journal.pone.0001975 18398485 PMC2276866

[148] De Maria A, Fogli M, Costa P, Murdaca G, Puppo F, Mavilio D, Moretta A, Moretta L The impaired NK cell cytolytic function in viremic HIV-1 infection is associated with a reduced surface expression of natural cytotoxicity receptors (NKp46, NKp30 and NKp44). Eur J Immunol. 2003;33(9):2410–8. PubMed PMID: 12938217. doi: 10.1002/eji.200324141 12938217

[149] Schwartz O Endocytosis of major histocompatibility complex class I molecules is induced by the HIV-1 Nef protein. Nat Med. 1996;2(3):338–42. PubMed PMID: 8612235. 8612235 10.1038/nm0396-338

[150] Cerboni C, Neri F, Casartelli N, Zingoni A, Cosman D, Rossi P, Santoni A, Doria M Human immunodeficiency virus 1 Nef protein downmodulates the ligands of the activating receptor NKG2D and inhibits natural killer cell-mediated cytotoxicity. J Gen Virol. 2007;88(Pt 1):242–50. PubMed PMID: 17170457. doi: 10.1099/vir.0.82125-0 17170457

[151] Galaski J, Ahmad F, Tibroni N, Pujol FM, Müller B, Schmidt RE, Fackler OT Cell Surface Downregulation of NK Cell Ligands by Patient-Derived HIV-1 Vpu and Nef Alleles. J Acquir Immune Defic Syndr. 2016;72(1):1–10. PubMed PMID: 26656785. doi: 10.1097/QAI.0000000000000917 26656785

[152] Parsons MS, Richard J, Lee WS, Vanderven H, Grant MD, Finzi A, Kent SJ NKG2D acts as a co-receptor for natural killer cell-mediated anti-HIV-1 antibody-dependent cellular cytotoxicity. AIDS Res Hum Retroviruses. 2016 PubMed PMID: 27487965. doi: 10.1089/AID.2016.0099 27487965

[153] Tomescu C, Mavilio D, Montaner LJ Lysis of HIV-1-infected autologous CD4+ primary T cells by interferon-alpha-activated NK cells requires NKp46 and NKG2D. AIDS. 2015;29(14):1767–73. PubMed PMID: 26372382. Pubmed Central PMCID: 4571461. doi: 10.1097/QAD.0000000000000777 26372382 PMC4571461

[154] Bisio F, Bozzano F, Marras F, Di Biagio A, Moretta L, De Maria A Successfully treated HIV-infected patients have differential expression of NK cell receptors (NKp46 and NKp30) according to AIDS status at presentation. Immunol Lett. 2013;152(1):16–24. PubMed PMID: 23538009. doi: 10.1016/j.imlet.2013.03.003 23538009

[155] Shah AH, Sowrirajan B, Davis ZB, Ward JP, Campbell EM, Planelles V, Barker E Degranulation of natural killer cells following interaction with HIV-1-infected cells is hindered by downmodulation of NTB-A by Vpu. Cell Host Microbe. 2010;8(5):397–409. PubMed PMID: 21075351. Pubmed Central PMCID: 3005698. doi: 10.1016/j.chom.2010.10.008 21075351 PMC3005698

[156] Brunetta E, Fogli M, Varchetta S, Bozzo L, Hudspeth KL, Marcenaro E, Moretta A, Mavilio D The decreased expression of Siglec-7 represents an early marker of dysfunctional natural killer-cell subsets associated with high levels of HIV-1 viremia. Blood. 2009;114(18):3822–30. PubMed PMID: 19710502. Pubmed Central PMCID: 2773483. doi: 10.1182/blood-2009-06-226332 19710502 PMC2773483

[157] Björkström NK, Svensson A, Malmberg K-J, Eriksson K, Ljunggren H-G Characterization of Natural Killer Cell Phenotype and Function during Recurrent Human HSV-2 Infection. Zimmer J, editor. PLoS ONE. 2011;6(11):e27664 PubMed PMID: 22110712. Pubmed Central PMCID: 3216993. doi: 10.1371/journal.pone.0027664 22110712 PMC3216993

[158] Campbell TM, McSharry BP, Steain M, Slobedman B, Abendroth A Varicella-Zoster Virus and Herpes Simplex Virus 1 Differentially Modulate NKG2D Ligand Expression during Productive Infection. Hutt-Fletcher L, editor. Journal of Virology. 2015;89(15):7932–43. PubMed PMID: 25995251. Pubmed Central PMCID: 4505661. doi: 10.1128/JVI.00292-15 25995251 PMC4505661

[159] Ishido S, Choi JK, Lee BS, Wang C, DeMaria M, Johnson RP, Cohen GB, Jung JU Inhibition of natural killer cell-mediated cytotoxicity by Kaposi's sarcoma-associated herpesvirus K5 protein. Immunity. 2000;13(3):365–74. PubMed PMID: 11021534. 11021534 10.1016/s1074-7613(00)00036-4

[160] Bjorkstrom NK, Lindgren T, Stoltz M, Fauriat C, Braun M, Evander M, Michaelsson J, Malmberg K-J, Klingstrom J, Ahlm C, Ljunggren HG Rapid expansion and long-term persistence of elevated NK cell numbers in humans infected with hantavirus. J Exp Med. 2011;208(1):13–21. PubMed PMID: 21173105. Pubmed Central PMCID: 3023129. doi: 10.1084/jem.20100762 21173105 PMC3023129

[161] Petitdemange C, Wauquier N, Devilliers H, Yssel H, Mombo I, Caron M, Nkoghé D, Debré P, Leroy E, Vieillard V Longitudinal Analysis of Natural Killer Cells in Dengue Virus-Infected Patients in Comparison to Chikungunya and Chikungunya/Dengue Virus-Infected Patients. PLoS Negl Trop Dis. 2016;10(3):e0004499 PubMed PMID: 26938618. Pubmed Central PMCID: 4777550. doi: 10.1371/journal.pntd.0004499 26938618 PMC4777550

[162] Beltrán D, Lopez-Vergès S NK Cells During Dengue Disease and Their Recognition of Dengue Virus-Infected Cells. Front Immunol. Frontiers; 2014;5(9599):192. 24829565 10.3389/fimmu.2014.00192 PMC4017149

[163] Hershkovitz O, Rosental B, Rosenberg LA, Navarro-Sanchez ME, Jivov S, Zilka A, Gershoni-Yahalom O, Brient-Litzler E, Bedouelle H, Ho JW, Campbell KS, Rager-Zisman B, Despres P, Porgador A NKp44 Receptor Mediates Interaction of the Envelope Glycoproteins from the West Nile and Dengue Viruses with NK Cells. J Immunol. 2009;183(4):2610–21. PubMed PMID: 19635919. Pubmed Central PMCID: 2768489. doi: 10.4049/jimmunol.0802806 19635919 PMC2768489

